# Supplementary material for: Unilateral cross-feeding constrains adaptive evolution, even in the producer without direct fitness effects
Source: Mol Biol Evol. 2026 Oct 21;43(11):msag263. doi: 10.1093/molbev/msag263 (PMC13733659; doi:10.1093/molbev/msag263)
Supplement: Supplementary file 1 [file msag263_supplementary_data.zip › MBE_SupplementaryMaterials_Final_20261015.docx]

**Supplementary Materials:** Unilateral cross-feeding constrains adaptive evolution, even in the producer without direct fitness effects.

**Supplementary Figures**

**Figure S1.** Population density (CFU mL^-1^) trajectories of six replicates of *A. johnsonii* and *P. putida* ancestors grown in monoculture (left panel) and co-culture (right panel) over four days of serial transfers.

**Figure S2.** (A) Parameter precision, shown as the mean within-group coefficient of variation (CV = SD/mean) of each fitted parameter across clones, averaged over all population × generation groups. The half-saturation constant (*K_m_*) is approximately five-fold more variable (CV ≈ 0.60) than the maximum growth rate (*µ_max_*), yield, and maximum uptake rate (*V_max_*) (CV ≈ 0.12–0.14). This indicates that *Km* is weakly constrained at the single-clone level, whereas the other parameters are well determined. (B) Population-level *K_m_* (mM) at generations 0, 400, and 800 in monoculture (light blue) and co-culture (dark blue). Points represent the mean of replicate-population means; error bars indicate 95% confidence intervals obtained by bootstrap resampling of populations (10,000 iterations). While *K_m_* declines under co-culture conditions, it remains stable in monoculture; the two conditions differ significantly at generation 800 (Welch's t-test, P = 0.012), and their confidence intervals do not overlap. Averaging across clones and replicate populations yields precise condition-level estimates despite the single-clone uncertainty shown in (A).

**Figure S3.** Half-saturation constant (*K_m_*) and maximum uptake rate (*V_max_*) for monoculture (left panel) and co-culture (right panel) evolved *P. putida.*

**Figure S4.** Heat map showing the frequency of nonsynonymous mutations (genes = rows) across generations in populations of *A. johnsonii* (red) and *P. putida* (blue). Six replicate monocultures (Aj1-Aj6 and PP1-PP6) and co-cultures (Co1-Co6) are shown. Only genes with mutations at frequencies above 0.1 are shown. The frequency of each mutation in the population is shown by different intensities of blue.

**Figure S5.** (A) Map of 72-k composite genomic island identified at the tmRNA ssrA locus of *A. johnsonii* C6. Island 1 (coordinates 856,233-861,527; 5.3 kb) contains phage-related genes, including a YqaJ viral recombinase. Island 2 (coordinates 889,088-900,395; 11.3 kb) contains an OmpA family protein and a large BapA/Bap family biofilm-associated protein. Island 3 (coordinates 908,174-921,589; 13.4 kb) contains metabolic genes including oxidoreductases, dehydrogenases, and an MFS transporter. Direct repeats (16 bp) flanking the region are indicated. (B) GC content variation across the 72-kb deleted region, with genomic islands highlighted. Compositional analysis revealed that the combined genomic islands exhibited significantly elevated GC content (43.0%) compared to the overall extracted region (40.1%) and flanking sequences (χ² = 80.3, p < 0.0001), consistent with horizontal acquisition from a donor organism with higher genomic GC content than *A. johnsonii* C6. Island 3 showed the strongest signature of foreign origin with 45.2% GC content. GC skew analysis showed relatively consistent values across the region (mean = 0.101 ± 0.042), suggesting the island had undergone amelioration toward host codon usage patterns over evolutionary time.

**Figure S6.** Selective sweeps of mutations reaching over 80% frequency in populations of *P. putida* (left panel) and *A. johnsonii* (right panel). Six replicate monocultures (Mono1-Mono6) and co-cultures (Co1-Co6) are shown.

**Figure S7.** Growth dynamics of three co-cultures revived from generation 6, 400, and 800 timepoints. Monocultures were derived from co-cultures (Co1 to Co3) using antibiotics. The area under the curve (AUC) was used to assess the growth of each species under each condition and across generations (Table S7). *P. putida* exhibited strong and consistent co-culture benefits across all generations and experimental lines (9/9 comparisons significant after FDR correction, p < 0.024 for all). Mean co-culture advantage ranged from +2.19 AUC units at 6 generations to +4.89 at 800 generations, representing substantial growth enhancement. *A. johnsonii* showed no significant co-culture effect at any generation (0/9 comparisons significant after FDR correction, p > 0.17 for all). Mean differences were near zero or slightly negative across all time points.

**Supplementary Tables**

**Table S1.** Resource use traits fold-change differences between monocultures and co-cultures for *Pseudomonas putida*. Values represent fold-change relative to the ancestor. Statistical comparisons by Welch’s *t*-test.

| **Trait** | **Generation** | ***n*_mono_** | ***n*_co_** | **Mean_mono_** | **SE_mono_** | **Mean_co_** | **SE_co_** | **Difference** | ***t*** | ***df*** | ***P*** | **Cohen’s *d*** |  |
| --- | --- | --- | --- | --- | --- | --- | --- | --- | --- | --- | --- | --- | --- |
| Yield | 400 | 5 | 6 | 2.882 | 0.071 | 2.221 | 0.262 | 0.661 | 2.431 | 9 | 0.053 | 1.347 | — |
| Yield | 800 | 5 | 6 | 3.186 | 0.145 | 2.469 | 0.131 | 0.717 | 3.674 | 9 | 0.006 | 2.228 | ** |
| *μ*_max_ | 400 | 5 | 6 | 2.834 | 0.037 | 2.141 | 0.271 | 0.693 | 2.536 | 9 | 0.050 | 1.393 | — |
| *μ*_max_ | 800 | 5 | 6 | 3.037 | 0.122 | 2.336 | 0.147 | 0.701 | 3.677 | 9 | 0.005 | 2.166 | ** |
| *K* | 400 | 5 | 6 | 0.973 | 0.084 | 0.771 | 0.027 | 0.203 | 2.288 | 9 | 0.073 | 1.500 | — |
| *K* | 800 | 5 | 6 | 1.018 | 0.091 | 0.648 | 0.034 | 0.370 | 3.823 | 9 | 0.012 | 2.489 | * |
| *V*_max_ | 400 | 5 | 6 | 1.009 | 0.026 | 0.930 | 0.017 | 0.079 | 2.567 | 9 | 0.036 | 1.603 | * |
| *V*_max_ | 800 | 5 | 6 | 1.026 | 0.026 | 0.913 | 0.019 | 0.113 | 3.502 | 9 | 0.009 | 2.173 | ** |

*Note:* μ_max_ = maximum growth rate; *K* = half-saturation constant; *V*_max_ = maximum uptake rate. Mono = monoculture; Co = coculture.

***** *P* < 0.05; ****** *P* < 0.01; — not significant.

**Table S2.** Pairwise comparisons of resource use traits across generations. A separate file (.xlsx) is provided.

**Table S3.** *de novo* mutations in populations and clones identified using *breseq.* A separate file (.xlsx) is provided.

**Table S4.** Nucleotide diversity ratios (*πN/πS*) for evolved populations of *P. putida* and *A. johnsonii* at generation 800.

| Species | Condition | nₙ | n_s_ | ΣπN | ΣπS | πN | πS | πN/πS |
| --- | --- | --- | --- | --- | --- | --- | --- | --- |
| *A. johnsonii* | Co-culture | 97 | 95 | 8.338 | 7.843 | 0.0860 | 0.0826 | 1.041† |
|  | Monoculture | 99 | 61 | 14.939 | 4.552 | 0.1509 | 0.0746 | 2.022 |
| *P. putida* | Co-culture | 11 | 10 | 5.490 | 6.620 | 0.4991 | 0.6620 | 0.754 |
|  | Monoculture | 343 | 230 | 29.610 | 13.820 | 0.0863 | 0.0601 | 1.437 |

nₙ and n_s_ are the numbers of nonsynonymous and neutral (synonymous + noncoding) sites; ΣπN and ΣπS are the summed per-site derived allele frequencies; *πN* and *πS* are the corresponding means (Σπ/n); *πN/πS* is their ratio.

† *A. johnsonii* co-culture values are shown after removal of all mutation calls at *gyrB*, *rpoB*, and *rpoC*, which were identified as cross-mapping artifacts (see Materials and Methods). The *A. johnsonii* monoculture and both *P. putida* rows are unchanged from the original analysis.

**Table S5.** Nucleotide diversity ratios (*πN/πS*) with 95% bootstrap confidence intervals and permutation-test support for the monoculture–co-culture difference, in evolved populations of *P. putida* and *A. johnsonii* at generation 800.

| **Species** | **Condition** | **πN/πS (95% CI)** | **Difference** |
| --- | --- | --- | --- |
| *A. johnsonii* | Monoculture | 2.02 [1.25, 3.07] | *p*-value = 0.036 |
|  | Co-culture | 1.04 [0.74, 1.45] † |  |
| *P. putida* | Monoculture | 1.44 [1.20, 1.72] | *p*-value = 0.42 (n.s.) |
|  | Co-culture | 0.75 [0.37, 1.46] |  |

95% confidence intervals were obtained by site-level bootstrap resampling of mutations (10,000 iterations). The monoculture–co-culture difference in *πN/πS* was assessed by a two-sided permutation test (10,000 permutations of treatment labels). The neutral reference class comprises synonymous and noncoding sites.

† *A. johnsonii* co-culture values are shown after removal of all mutation calls at *gyrB*, *rpoB*, and *rpoC*, which were identified as cross-mapping artifacts (see Materials and Methods); the *A. johnsonii* monoculture value and both *P. putida* values are unchanged from the original analysis.

**Table S6.** Predicted genomic islands in *A. johnsonii* C6 identified by IslandViewer 4. Gene content for each island is listed with locus tags, coordinates, and predicted products.

| **Island** | **Coordinates** | **Length (bp)** | **Prediction method** | **Locus tag** | **Start** | **End** | **Strand** | **Product** |
| --- | --- | --- | --- | --- | --- | --- | --- | --- |
| 1 | 856,233–861,527 | 5,294 | Predicted by ≥1 method | ACVIAJ_000809 | 856,233 | 856,508 | − | hypothetical protein |
|  |  |  |  | ACVIAJ_000810 | 857,335 | 857,652 | + | hypothetical protein |
|  |  |  |  | ACVIAJ_000811 | 857,691 | 858,737 | + | DUF932 domain-containing protein |
|  |  |  |  | ACVIAJ_000812 | 858,887 | 859,930 | + | YqaJ viral recombinase family protein |
|  |  |  |  | ACVIAJ_000813 | 860,048 | 860,977 | + | hydrolase or metal-binding protein |
|  |  |  |  | ACVIAJ_000814 | 861,045 | 861,275 | + | helix-turn-helix transcriptional regulator |
|  |  |  |  | ACVIAJ_000815 | 861,348 | 861,527 | + | hypothetical protein |
| 2 | 889,088–900,395 | 11,307 | Predicted by ≥1 method | ACVIAJ_000830 | 889,088 | 889,465 | + | helix-turn-helix domain-containing protein |
|  |  |  |  | ACVIAJ_000831 | 889,472 | 890,296 | − | hypothetical protein |
|  |  |  |  | ACVIAJ_000832 | 891,276 | 892,127 | + | OmpA family protein |
|  |  |  |  | ACVIAJ_000833 | 892,170 | 900,395 | + | BapA/Bap/LapF family prefix-like domain-containing protein |
| 3 | 908,174–921,589 | 13,415 | Predicted by ≥1 method | ACVIAJ_000840 | 908,174 | 908,473 | + | recombinase-like helix-turn-helix domain-containing protein |
|  |  |  |  | ACVIAJ_000841 | 908,489 | 909,553 | + | Rieske 2Fe-2S domain-containing protein |
|  |  |  |  | ACVIAJ_000842 | 909,546 | 910,310 | + | SDR family oxidoreductase |
|  |  |  |  | ACVIAJ_000843 | 910,321 | 911,250 | + | VOC family protein |
|  |  |  |  | ACVIAJ_000844 | 911,276 | 912,484 | + | NAD(P)/FAD-dependent oxidoreductase |
|  |  |  |  | ACVIAJ_000845 | 912,525 | 913,748 | + | MFS transporter |
|  |  |  |  | ACVIAJ_000846 | 913,914 | 914,435 | + | cupin domain-containing protein |
|  |  |  |  | ACVIAJ_000847 | 914,451 | 915,302 | + | alpha/beta fold hydrolase |
|  |  |  |  | ACVIAJ_000848 | 915,312 | 916,106 | + | SDR family oxidoreductase |
|  |  |  |  | ACVIAJ_000849 | 916,116 | 916,907 | + | aspartate dehydrogenase |
|  |  |  |  | ACVIAJ_000850 | 916,927 | 918,393 | + | aldehyde dehydrogenase |
|  |  |  |  | ACVIAJ_000851 | 918,442 | 920,082 | + | thiamine pyrophosphate-binding protein |
|  |  |  |  | ACVIAJ_000852 | 920,390 | 921,589 | + | hypothetical protein |

*Note:* Islands were predicted by at least one of the integrated methods in IslandViewer 4 (IslandPath-DIMOB, SIGI-HMM, or IslandPick). Coordinates refer to positions in the complete chromosome (GenBank accession PRJNA1339041). Strand: +, sense; −, antisense.

**Table S7.** Significantly enriched Gene Ontology (GO) pathways among genes harboring mutations in evolved populations of *Pseudomonas putida* and *Acinetobacter johnsonii*. Enrichment was assessed by Fisher’s exact test with FDR correction (Benjamini–Hochberg).

| **Condition** | **Species** | **GO pathway** | **log_2_(fold enrichment)** | **FDR** |
| --- | --- | --- | --- | --- |
| Coculture | *P. putida* | Biological regulation | 2.149 | 3.90 × 10^‒2^ |
|  |  | Peptidyl-amino acid modification | 4.540 | 3.62 × 10^‒2^ |
|  |  | Peptidyl-histidine modification | 5.083 | 2.39 × 10^‒2^ |
|  |  | Peptidyl-histidine phosphorylation | 5.083 | 2.39 × 10^‒2^ |
|  |  | Protein metabolic process | 2.925 | 3.90 × 10^‒2^ |
|  |  | Protein modification process | 3.911 | 4.46 × 10^‒2^ |
|  |  | Protein phosphorylation | 4.816 | 2.96 × 10^‒2^ |
|  |  | Regulation of biological process | 2.257 | 3.62 × 10^‒2^ |
|  |  | Regulation of cell motility | 6.442 | 6.36 × 10^‒3^ |
|  |  | Regulation of locomotion | 6.322 | 6.36 × 10^‒3^ |
| Monoculture | *P. putida* | Bacterial-type flagellum assembly | 6.480 | 5.45 × 10^‒3^ |
|  |  | Bacterial-type flagellum organization | 5.764 | 7.42 × 10^‒3^ |
|  |  | Cell projection assembly | 5.334 | 1.06 × 10^‒2^ |
|  |  | Cell projection organization | 4.930 | 1.72 × 10^‒2^ |
|  |  | Cellular component assembly | 3.560 | 4.18 × 10^‒2^ |
|  |  | Non-membrane-bounded organelle assembly | 5.764 | 7.42 × 10^‒3^ |
|  |  | Nucleic acid-templated transcription | 4.288 | 3.19 × 10^‒2^ |
|  |  | Organelle assembly | 5.764 | 7.42 × 10^‒3^ |
|  |  | Organelle organization | 4.002 | 3.19 × 10^‒2^ |
|  |  | Regulation of cell adhesion | 7.288 | 3.19 × 10^‒2^ |
|  |  | Regulation of cell motility | 6.349 | 2.74 × 10^‒4^ |
|  |  | Regulation of cell‑substrate adhesion | 7.703 | 3.19 × 10^‒2^ |
|  |  | Regulation of cellular component biogenesis | 6.966 | 3.71 × 10^‒2^ |
|  |  | Regulation of cellular response to stress | 6.480 | 4.18 × 10^‒2^ |
|  |  | Regulation of chemotaxis | 7.288 | 3.19 × 10^‒2^ |
|  |  | Regulation of locomotion | 6.229 | 2.74 × 10^‒4^ |
|  |  | Regulation of response to external stimulus | 6.480 | 4.18 × 10^‒2^ |
|  |  | Regulation of response to oxidative stress | 7.288 | 3.19 × 10^‒2^ |
|  |  | Regulation of secondary metabolic process | 5.644 | 7.42 × 10^‒3^ |
|  |  | Regulation of secondary metabolite biosynthetic process | 5.644 | 7.42 × 10^‒3^ |
|  |  | RNA biosynthetic process | 4.138 | 3.19 × 10^‒2^ |
|  |  | Transcription, DNA-templated | 4.288 | 3.19 × 10^‒2^ |
| Coculture | *A. johnsonii* | Acyltransferase activity, transferring groups other than amino-acyl groups | 7.040 | 7.58 × 10^‒3^ |
| Monoculture | *A. johnsonii* | Acyltransferase activity, transferring groups other than amino-acyl groups | 5.455 | 4.52 × 10^‒2^ |

*Note:* log_2_(fold enrichment) indicates the magnitude of overrepresentation relative to the genomic background. Only pathways with FDR < 0.05 are shown. Rows are sorted alphabetically within each condition × species group.

**Table S8**. Per-population lineage counts and lineage divergence for *P. putida* evolved in monoculture and co-culture.

| **Population** | **Evolved lineages** | **Max mutations per lineage** |
| --- | --- | --- |
| Monoculture 1 | 12 | 6 |
| Monoculture 2 | 10 | 7 |
| Monoculture 3 | 6 | 4 |
| Monoculture 4 | 5 | 6 |
| Monoculture 6 | 8 | 5 |
| Monoculture mean ± SD | 8.2 ± 2.9 | 5.6 ± 1.1 |
| Co-culture 1 | 7 | 4 |
| Co-culture 2 | 5 | 4 |
| Co-culture 3 | 6 | 6 |
| Co-culture 4 | 5 | 2 |
| Co-culture 5 | 4 | 4 |
| Co-culture 6 | 4 | 5 |
| Co-culture mean ± SD | 5.2 ± 1.2 | 4.2 ± 1.3 |

Evolved lineages: number of distinct reconstructed genotypes per population, excluding the ancestral background. Max mutations per lineage: the largest number of accumulated mutations in any single lineage.

**Table S9.** Calculated area under the curve (AUC)

| **Generation** | **Species** | **Experimental line** | **Replicate** | **AUC_Co_** | **AUC_Mono_** | **Partner's effect**  **(AUC_Co_ - AUC_Mono_)** |
| --- | --- | --- | --- | --- | --- | --- |
| 6 | *A. johnsonii* | co1 | 1 | 23.635 | 23.609 | 0.027 |
| 6 | *A. johnsonii* | co1 | 2 | 23.589 | 23.151 | 0.438 |
| 6 | *A. johnsonii* | co1 | 3 | 23.310 | 23.385 | -0.075 |
| 6 | *A. johnsonii* | co2 | 1 | 23.642 | 23.259 | 0.383 |
| 6 | *A. johnsonii* | co2 | 2 | 23.410 | 23.347 | 0.063 |
| 6 | *A. johnsonii* | co2 | 3 | 23.186 | 23.257 | -0.071 |
| 6 | *A. johnsonii* | co3 | 1 | 23.506 | 22.988 | 0.518 |
| 6 | *A. johnsonii* | co3 | 2 | 23.584 | 23.402 | 0.182 |
| 6 | *A. johnsonii* | co3 | 3 | 23.041 | 23.250 | -0.209 |
| 6 | *P. putida* | co1 | 1 | 18.847 | 17.391 | 1.456 |
| 6 | *P. putida* | co1 | 2 | 18.965 | 16.701 | 2.264 |
| 6 | *P. putida* | co1 | 3 | 18.705 | 15.840 | 2.865 |
| 6 | *P. putida* | co2 | 1 | 18.900 | 16.634 | 2.266 |
| 6 | *P. putida* | co2 | 2 | 18.786 | 16.350 | 2.436 |
| 6 | *P. putida* | co2 | 3 | 18.804 | 16.396 | 2.408 |
| 6 | *P. putida* | co3 | 1 | 18.739 | 16.652 | 2.087 |
| 6 | *P. putida* | co3 | 2 | 18.782 | 15.994 | 2.788 |
| 6 | *P. putida* | co3 | 3 | 18.801 | 16.253 | 2.548 |
| 400 | *A. johnsonii* | co1 | 1 | 24.397 | 24.481 | -0.084 |
| 400 | *A. johnsonii* | co1 | 2 | 24.377 | 24.265 | 0.111 |
| 400 | *A. johnsonii* | co1 | 3 | 24.059 | 24.172 | -0.114 |
| 400 | *A. johnsonii* | co2 | 1 | 24.329 | 24.330 | 0.000 |
| 400 | *A. johnsonii* | co2 | 2 | 24.310 | 24.413 | -0.103 |
| 400 | *A. johnsonii* | co2 | 3 | 24.080 | 24.292 | -0.212 |
| 400 | *A. johnsonii* | co3 | 1 | 24.610 | 24.046 | 0.565 |
| 400 | *A. johnsonii* | co3 | 2 | 24.319 | 24.393 | -0.074 |
| 400 | *A. johnsonii* | co3 | 3 | 23.918 | 24.244 | -0.326 |
| 400 | *P. putida* | co1 | 1 | 18.433 | 16.631 | 1.802 |
| 400 | *P. putida* | co1 | 2 | 18.932 | 16.752 | 2.180 |
| 400 | *P. putida* | co1 | 3 | 18.998 | 16.336 | 2.662 |
| 400 | *P. putida* | co2 | 1 | 20.196 | NA | NA |
| 400 | *P. putida* | co2 | 2 | 20.396 | 16.495 | 3.902 |
| 400 | *P. putida* | co2 | 3 | 20.134 | 16.327 | 3.807 |
| 400 | *P. putida* | co3 | 1 | 19.017 | 16.651 | 2.366 |
| 400 | *P. putida* | co3 | 2 | 18.946 | 16.664 | 2.282 |
| 400 | *P. putida* | co3 | 3 | 18.724 | 16.393 | 2.332 |
| 800 | *A. johnsonii* | co1 | 1 | 23.653 | 24.377 | -0.724 |
| 800 | *A. johnsonii* | co1 | 2 | 23.647 | 24.045 | -0.397 |
| 800 | *A. johnsonii* | co1 | 3 | 23.518 | 23.682 | -0.164 |
| 800 | *A. johnsonii* | co2 | 1 | 23.468 | 24.241 | -0.773 |
| 800 | *A. johnsonii* | co2 | 2 | 23.896 | 24.102 | -0.206 |
| 800 | *A. johnsonii* | co2 | 3 | 23.541 | 24.053 | -0.511 |
| 800 | *A. johnsonii* | co3 | 1 | 24.369 | 24.257 | 0.112 |
| 800 | *A. johnsonii* | co3 | 2 | 24.282 | 24.028 | 0.255 |
| 800 | *A. johnsonii* | co3 | 3 | 23.618 | 24.052 | -0.434 |
| 800 | *P. putida* | co1 | 1 | 20.648 | 16.387 | 4.261 |
| 800 | *P. putida* | co1 | 2 | 22.321 | 17.076 | 5.245 |
| 800 | *P. putida* | co1 | 3 | 21.426 | 17.667 | 3.759 |
| 800 | *P. putida* | co2 | 1 | 20.834 | 16.230 | 4.604 |
| 800 | *P. putida* | co2 | 2 | 21.457 | 16.856 | 4.601 |
| 800 | *P. putida* | co2 | 3 | 21.469 | 16.004 | 5.466 |
| 800 | *P. putida* | co3 | 1 | 19.103 | 15.778 | 3.325 |
| 800 | *P. putida* | co3 | 2 | 19.966 | 16.791 | 3.175 |
| 800 | *P. putida* | co3 | 3 | 19.993 | 16.304 | 3.688 |

**Table S10.** Linear mixed-effects model results for the effect of generation on the difference in area under the curve (AUC) between coculture and monoculture conditions. Separate models were fit for *A. johnsonii* and *P. putida*. Values in parentheses are standard errors.

|  | ***A. johnsonii*** | ***P. putida*** |
| --- | --- | --- |
| **Model specification** |  |  |
| Formula | *Difference* ~ *Generation* + (1\|*Line*) | *Difference* ~ *Generation* + (1\|*Line*) |
| Observations | 27 | 26 |
| Experimental lines | 3 | 3 |
| **Fixed effects** |  |  |
| Intercept | 0.160 (0.095) | 2.172 (0.324) |
| Intercept *P* | 0.135 | 0.006 ** |
| Generation slope | −0.00057 (0.00016) | +0.00236 (0.00037) |
| Generation *P* | 0.002 ** | < 0.001 *** |
| **Random effects (σ²)** |  |  |
| Line (intercept) | 0.072 | 0.449 |
| Residual | 0.279 | 0.633 |
| **Interpretation** |  |  |
| Effect direction | Coculture benefit decreases over time | Coculture benefit increases over time |
| Rate of change | −0.00057 per generation | +0.00236 per generation |

*Note:* Experimental line was included as a random intercept. * *P* < 0.05; ** *P* < 0.01; *** *P* < 0.001.

**Table S11.** **Sequencing coverage and downsampling corrections for evolved populations.** The reference genome lengths used across all samples were 6,181,873 bp for *P. putida* KT2440 and 3,746,335 bp for *A. johnsonii* C6. The 'Passed Filters' corresponds to the percentage of reads passing the quality thresholds during the *breseq* -l 100 downsampling step. To determine the original, pre-downsampled coverage for each species (Estimated Original Coverage per Species), values were back-calculated by dividing the Post-Sampled Coverage (*fit mean* in *breseq*) by the sampling factor (Passed Filters divided by 100). We also report the raw sequencing coverage of the ancestral populations, which did not undergo downsampling corrections.

| **Sample ID** | **Target Species** | **Passed Filters** | **Post-Sampled Coverage (*fit mean*)*** | **Estimated Original Coverage per Species** |
| --- | --- | --- | --- | --- |
| Coculture1 T800 | *P. putida* | 58.8% | 97.5x | 165.8x |
|  | *A. johnsonii* | 60.8% | 92.7x | 152.5x |
| Coculture2 T800 | *P. putida* | 69.9% | 97.8x | 139.9x |
|  | *A. johnsonii* | 76.8% | 84.7x | 110.3x |
| Coculture3 T800 | *P. putida* | 87.9% | 99.4x | 113.1x |
|  | *A. johnsonii* | 68.9% | 85.8x | 124.5x |
| Coculture4 T800 | *P. putida* | 79.3% | 99.7x | 125.7x |
|  | *A. johnsonii* | 60.0% | 83.0x | 138.3x |
| Coculture5 T800 | *P. putida* | 82.0% | 98.4x | 120.0x |
|  | *A. johnsonii* | 63.3% | 91.9x | 145.2x |
| Coculture6 T800 | *P. putida* | 83.4% | 98.0x | 117.5x |
|  | *A. johnsonii* | 63.2% | 96.2x | 152.2x |
| Monoculture1 T800 | *P. putida* | 79.3% | 100.1x | 126.2x |
| Monoculture2 T800 | *P. putida* | 96.2% | 100.2x | 104.2x |
| Monoculture3 T800 | *P. putida* | 65.3% | 99.7x | 152.7x |
| Monoculture4 T800 | *P. putida* | 99.8% | 99.6x | 99.8x |
| Monoculture6 T800 | *P. putida* | 95.5% | 99.8x | 104.5x |
| Monoculture1 T800 | *A. johnsonii* | 71.4% | 102.4x | 143.4x |
| Monoculture2 T800 | *A. johnsonii* | 92.9% | 102.3x | 110.1x |
| Monoculture3 T800 | *A. johnsonii* | 99.9% | 86.7x | 86.8x |
| Monoculture4 T800 | *A. johnsonii* | 75.6% | 100.3x | 132.7x |
| Monoculture5 T800 | *A. johnsonii* | 59.3% | 100.4x | 169.3x |
| Monoculture6 T800 | *A. johnsonii* | 66.8% | 100.5x | 150.4x |
| Coculture1 T600 | *P. putida* | 70.2% | 99.4x | 141.6x |
|  | *A. johnsonii* | 66.5% | 91.4x | 137.4x |
| Coculture2 T600 | *P. putida* | 72.5% | 98.5x | 135.9x |
|  | *A. johnsonii* | 68.9% | 85.0x | 123.4x |
| Coculture3 T600 | *P. putida* | 90.4% | 98.1x | 108.5x |
|  | *A. johnsonii* | 67.0% | 82.3x | 122.8x |
| Coculture4 T600 | *P. putida* | 97.7% | 99.2x | 101.5x |
|  | *A. johnsonii* | 61.6% | 84.2x | 136.7x |
| Coculture5 T600 | *P. putida* | 81.2% | 98.7x | 121.6x |
|  | *A. johnsonii* | 95.8% | 88.3x | 92.2x |
| Coculture6 T600 | *P. putida* | 65.1% | 93.7x | 143.9x |
|  | *A. johnsonii* | 86.9% | 81.4x | 93.7x |
| Monoculture1 T600 | *P. putida* | 99.8% | 94.1x | 94.3x |
| Monoculture2 T600 | *P. putida* | 87.0% | 100.6x | 115.6x |
| Monoculture3 T600 | *P. putida* | 87.4% | 100.7x | 115.2x |
| Monoculture4 T600 | *P. putida* | 85.7% | 100.1x | 116.8x |
| Monoculture6 T600 | *P. putida* | 83.6% | 99.6x | 119.1x |
| Monoculture1 T600 | *A. johnsonii* | 65.3% | 102.5x | 157.0x |
| Monoculture2 T600 | *A. johnsonii* | 92.0% | 103.0x | 112.0x |
| Monoculture3 T600 | *A. johnsonii* | 52.8% | 100.6x | 190.6x |
| Monoculture4 T600 | *A. johnsonii* | 90.5% | 100.6x | 111.2x |
| Monoculture5 T600 | *A. johnsonii* | 99.9% | 59.6x | 59.7x |
| Monoculture6 T600 | *A. johnsonii* | 78.4% | 100.6x | 128.3x |
| Coculture1 T400 | *P. putida* | 87.9% | 101.9x | 115.9x |
|  | *A. johnsonii* | 92.4% | 93.3x | 101.0x |
| Coculture2 T400 | *P. putida* | 73.3% | 98.9x | 134.9x |
|  | *A. johnsonii* | 59.3% | 86.5x | 145.9x |
| Coculture3 T400 | *P. putida* | 75.6% | 99.5x | 131.6x |
|  | *A. johnsonii* | 58.5% | 91.1x | 155.7x |
| Coculture4 T400 | *P. putida* | 54.0% | 100.3x | 185.7x |
|  | *A. johnsonii* | 79.9% | 88.7x | 111.0x |
| Coculture5 T400 | *P. putida* | 83.6% | 99.4x | 118.9x |
|  | *A. johnsonii* | 56.4% | 84.4x | 149.6x |
| Coculture6 T400 | *P. putida* | 98.2% | 99.7x | 101.5x |
|  | *A. johnsonii* | 68.4% | 87.7x | 128.2x |
| Monoculture1 T400 | *P. putida* | 86.4% | 99.6x | 115.3x |
| Monoculture2 T400 | *P. putida* | 91.9% | 100.6x | 109.5x |
| Monoculture3 T400 | *P. putida* | 75.0% | 100.0x | 133.3x |
| Monoculture4 T400 | *P. putida* | 96.1% | 99.4x | 103.4x |
| Monoculture6 T400 | *P. putida* | 93.2% | 99.4x | 106.7x |
| Monoculture1 T400 | *A. johnsonii* | 91.2% | 100.9x | 110.6x |
| Monoculture2 T400 | *A. johnsonii* | 56.1% | 100.8x | 179.7x |
| Monoculture3 T400 | *A. johnsonii* | 96.0% | 100.6x | 104.8x |
| Monoculture4 T400 | *A. johnsonii* | 94.2% | 100.5x | 106.7x |
| Monoculture5 T400 | *A. johnsonii* | 77.7% | 100.7x | 129.6x |
| Monoculture6 T400 | *A. johnsonii* | 52.6% | 100.2x | 190.5x |
| Coculture1 T200 | *P. putida* | 22.0% | 100.8x | 458.2x |
|  | *A. johnsonii* | 27.6% | 100.2x | 363.0x |
| Coculture2 T200 | *P. putida* | 31.3% | 101.3x | 323.6x |
|  | *A. johnsonii* | 26.8% | 100.2x | 373.9x |
| Coculture3 T200 | *P. putida* | 17.8% | 100.8x | 566.3x |
|  | *A. johnsonii* | 29.9% | 100.1x | 334.8x |
| Coculture4 T200 | *P. putida* | 22.6% | 100.9x | 446.5x |
|  | *A. johnsonii* | 14.1% | 100.3x | 711.3x |
| Coculture5 T200 | *P. putida* | 17.4% | 100.8x | 579.3x |
|  | *A. johnsonii* | 14.8% | 100.1x | 676.4x |
| Coculture6 T200 | *P. putida* | 42.9% | 101.3x | 236.1x |
|  | *A. johnsonii* | 29.6% | 100.9x | 340.9x |
| Monoculture1 T200 | *P. putida* | 45.8% | 101.0x | 220.5x |
| Monoculture2 T200 | *P. putida* | 21.9% | 101.0x | 461.2x |
| Monoculture3 T200 | *P. putida* | 82.4% | 101.4x | 123.1x |
| Monoculture4 T200 | *P. putida* | 40.9% | 101.1x | 247.2x |
| Monoculture6 T200 | *P. putida* | 35.1% | 101.3x | 288.6x |
| Monoculture1 T200 | *A. johnsonii* | 23.1% | 100.3x | 434.2x |
| Monoculture2 T200 | *A. johnsonii* | 26.1% | 100.3x | 384.3x |
| Monoculture3 T200 | *A. johnsonii* | 34.8% | 100.2x | 287.9x |
| Monoculture4 T200 | *A. johnsonii* | 44.2% | 100.2x | 226.7x |
| Monoculture5 T200 | *A. johnsonii* | 49.3% | 100.3x | 203.5x |
| Monoculture6 T200 | *A. johnsonii* | 17.8% | 100.0x | 561.8x |
| Ancestral population | *P. putida* | 100.0% | NA | 714.1x |
| Ancestral population | *A. johnsonii* | 100.0% | NA | 638.8x |

***** Note on A. johnsonii metrics: The reported Post-Sampled Coverage represents the length-weighted average calculated all reference contigs/sequences, to prevent smaller elements with high copy numbers (e.g., the 9.3-kb plasmid sequence 2) from artificially inflating the reported sequence depth.
