# Supplementary material for: Unilateral cross-feeding constrains adaptive evolution, even in the producer without direct fitness effects
Source: Mol Biol Evol. 2026 Oct 21;43(11):msag263. doi: 10.1093/molbev/msag263 (PMC13733659; doi:10.1093/molbev/msag263)
Supplement: Supplementary file 1 [file msag263_supplementary_data.zip › Supplementary_FigureS1.pdf]

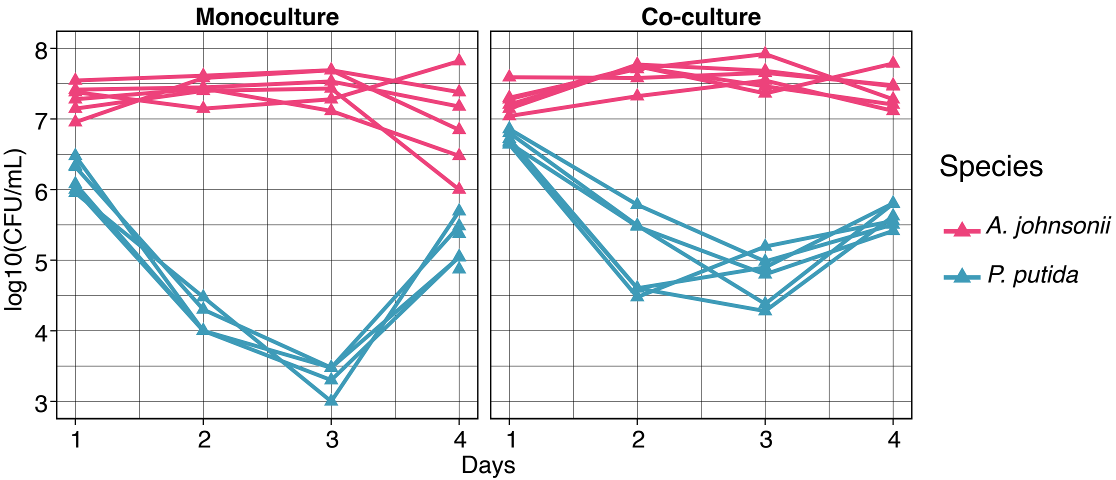
Monoculture
Co-culture
log10(CFU/mL)
8
7
6
5
4
3
1
2
3
4
Days
Species
A. johnsonii
P. putida
