# Supplementary material for: Unilateral cross-feeding constrains adaptive evolution, even in the producer without direct fitness effects
Source: Mol Biol Evol. 2026 Oct 21;43(11):msag263. doi: 10.1093/molbev/msag263 (PMC13733659; doi:10.1093/molbev/msag263)
Supplement: Supplementary file 1 [file msag263_supplementary_data.zip › Supplementary_FigureS2.pdf]

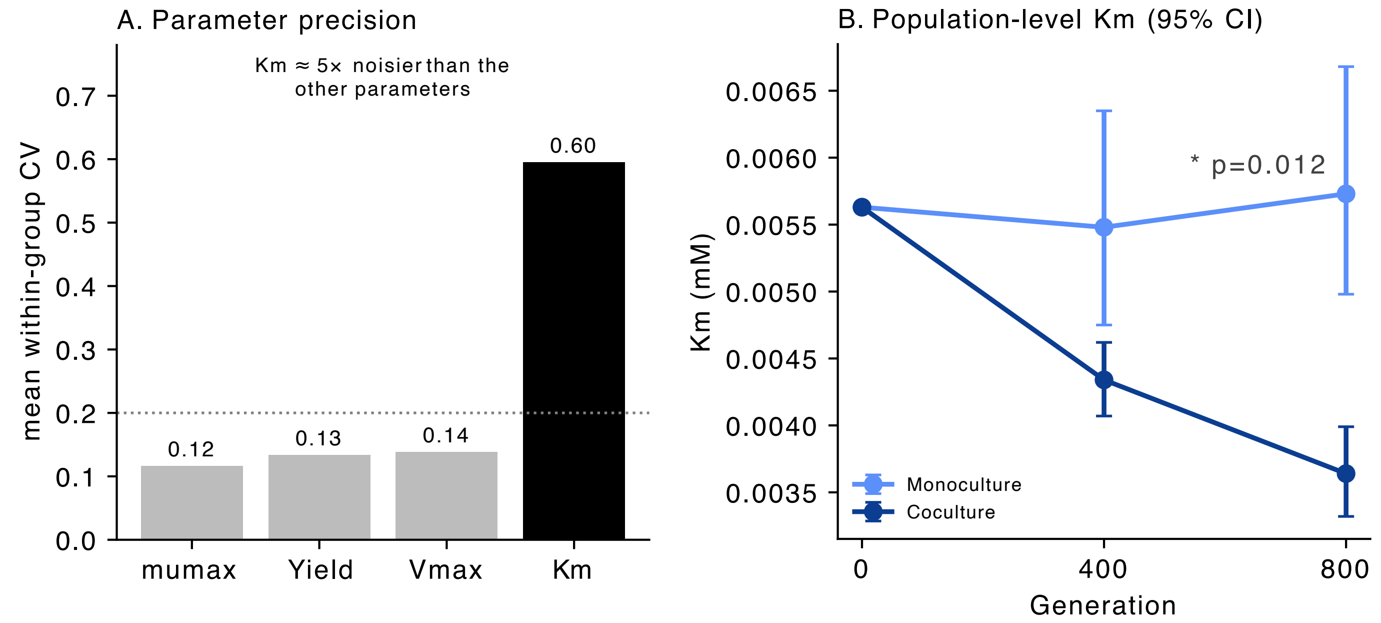
A. Parameter precision
Km ≈ 5× noisier than the other parameters
mean within-group CV
0.12
0.13
0.14
0.60
mumax
Yield
Vmax
Km
B. Population-level Km (95% CI)
Km (mM)
* p=0.012
Monoculture
Coculture
Generation
