# Supplementary material for: Unilateral cross-feeding constrains adaptive evolution, even in the producer without direct fitness effects
Source: Mol Biol Evol. 2026 Oct 21;43(11):msag263. doi: 10.1093/molbev/msag263 (PMC13733659; doi:10.1093/molbev/msag263)
Supplement: Supplementary file 1 [file msag263_supplementary_data.zip › Supplementary_FigureS3.pdf]

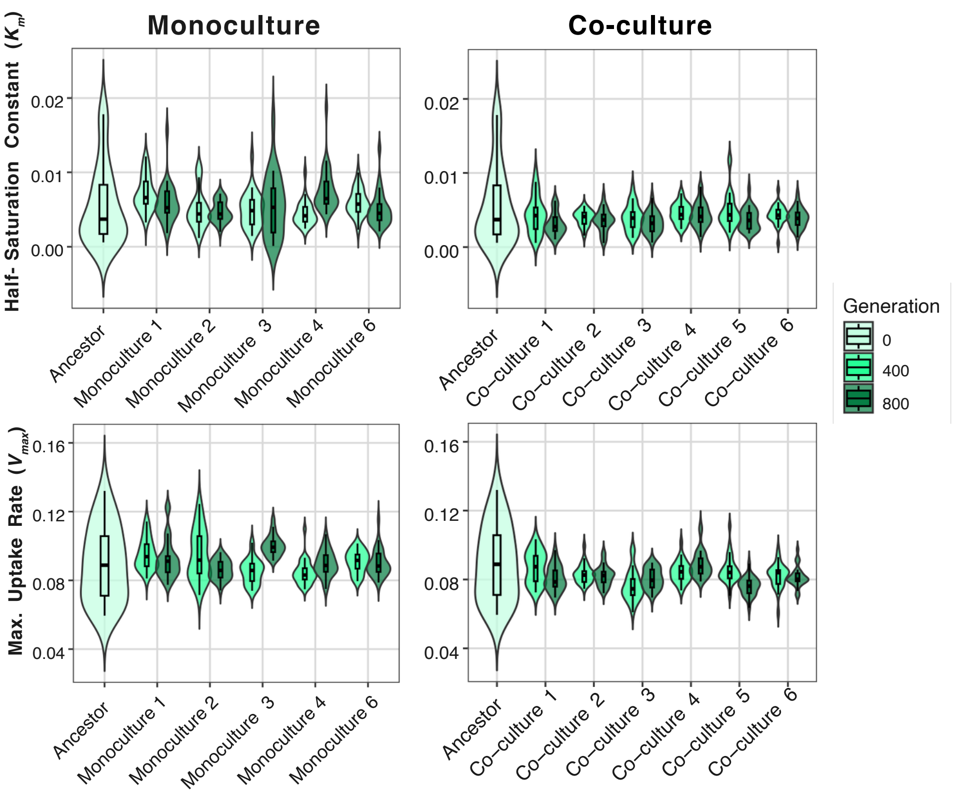

Monoculture
Co-culture
Half- Saturation Constant ($K_m$)
Max. Uptake Rate ($V_{max}$)
0.02
0.01
0.00
0.16
0.12
0.08
0.04
Ancestor
Monoculture 1
Monoculture 2
Monoculture 3
Monoculture 4
Monoculture 6
Co–culture 1
Co–culture 2
Co–culture 3
Co–culture 4
Co–culture 5
Co–culture 6
Generation
0
400
800
