# Supplementary material for: Unilateral cross-feeding constrains adaptive evolution, even in the producer without direct fitness effects
Source: Mol Biol Evol. 2026 Oct 21;43(11):msag263. doi: 10.1093/molbev/msag263 (PMC13733659; doi:10.1093/molbev/msag263)
Supplement: Supplementary file 1 [file msag263_supplementary_data.zip › Supplementary_FigureS4.pdf]

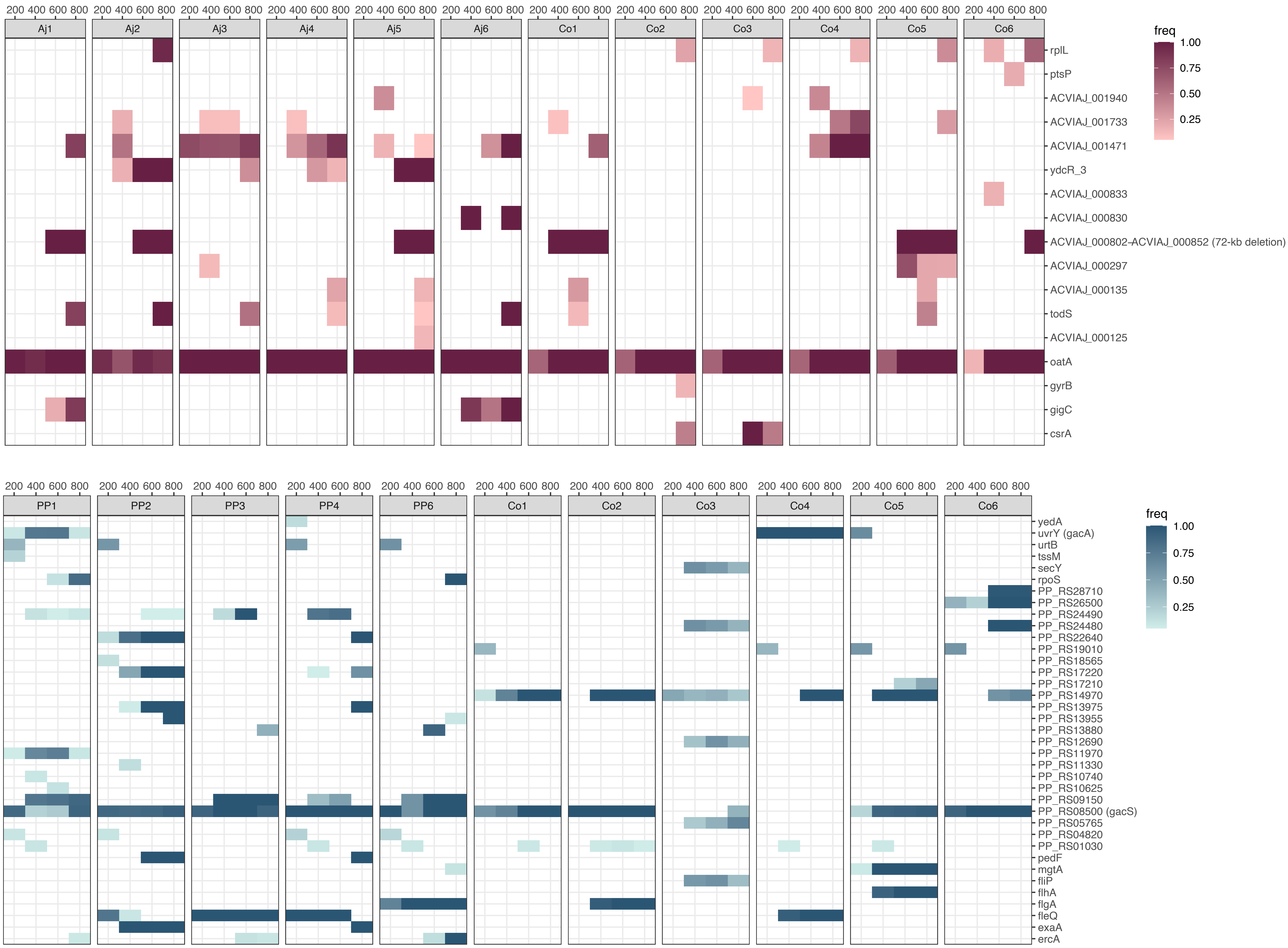

200 400 600 800
Aj1
Aj2
Aj3
Aj4
Aj5
Aj6
Co1
Co2
Co3
Co4
Co5
Co6
freq
1.00
0.75
0.50
0.25
rplL
ptsP
ACVIAJ_001940
ACVIAJ_001733
ACVIAJ_001471
ydcR_3
ACVIAJ_000833
ACVIAJ_000830
ACVIAJ_000802–ACVIAJ_000852 (72-kb deletion)
ACVIAJ_000297
ACVIAJ_000135
todS
ACVIAJ_000125
oatA
gyrB
gigC
csrA
PP1
PP2
PP3
PP4
PP6
yedA
uvrY (gacA)
urtB
tssM
secY
rpoS
PP_RS28710
PP_RS26500
PP_RS24490
PP_RS24480
PP_RS22640
PP_RS19010
PP_RS18565
PP_RS17220
PP_RS17210
PP_RS14970
PP_RS13975
PP_RS13955
PP_RS13880
PP_RS12690
PP_RS11970
PP_RS11330
PP_RS10740
PP_RS10625
PP_RS09150
PP_RS08500 (gacS)
PP_RS05765
PP_RS04820
PP_RS01030
pedF
mgtA
fliP
flhA
flgA
fleQ
exaA
ercA
