# Supplementary material for: Unilateral cross-feeding constrains adaptive evolution, even in the producer without direct fitness effects
Source: Mol Biol Evol. 2026 Oct 21;43(11):msag263. doi: 10.1093/molbev/msag263 (PMC13733659; doi:10.1093/molbev/msag263)
Supplement: Supplementary file 1 [file msag263_supplementary_data.zip › Supplementary_FigureS5.pdf]

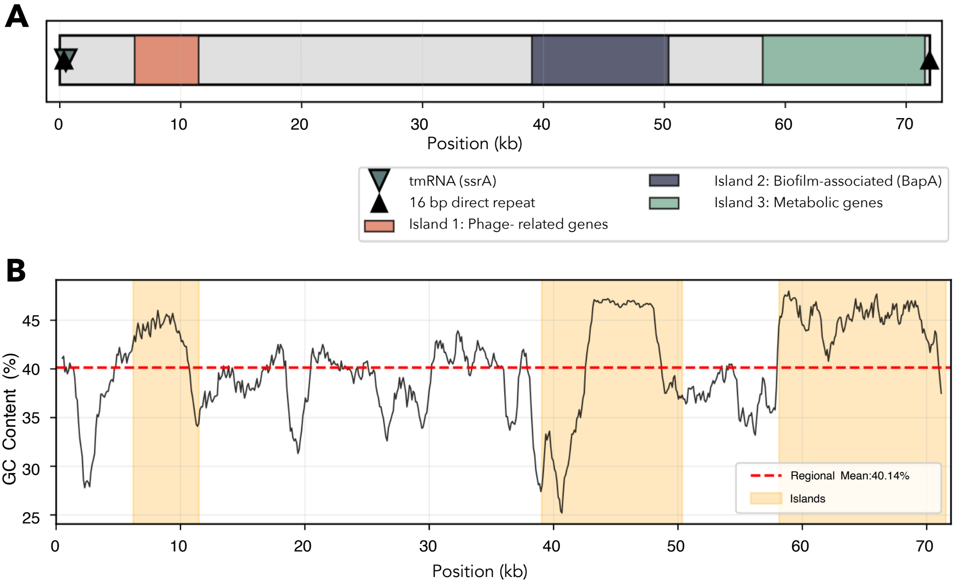
A
0
10
20
30
40
50
60
70
Position (kb)
tmRNA (ssrA)
16 bp direct repeat
Island 1: Phage- related genes
Island 2: Biofilm-associated (BapA)
Island 3: Metabolic genes
B
GC Content (%)
25
30
35
40
45
Regional Mean:40.14%
Islands
0
10
20
30
40
50
60
70
Position (kb)
