# Supplementary material for: Unilateral cross-feeding constrains adaptive evolution, even in the producer without direct fitness effects
Source: Mol Biol Evol. 2026 Oct 21;43(11):msag263. doi: 10.1093/molbev/msag263 (PMC13733659; doi:10.1093/molbev/msag263)
Supplement: Supplementary file 1 [file msag263_supplementary_data.zip › Supplementary_FigureS6.pdf]

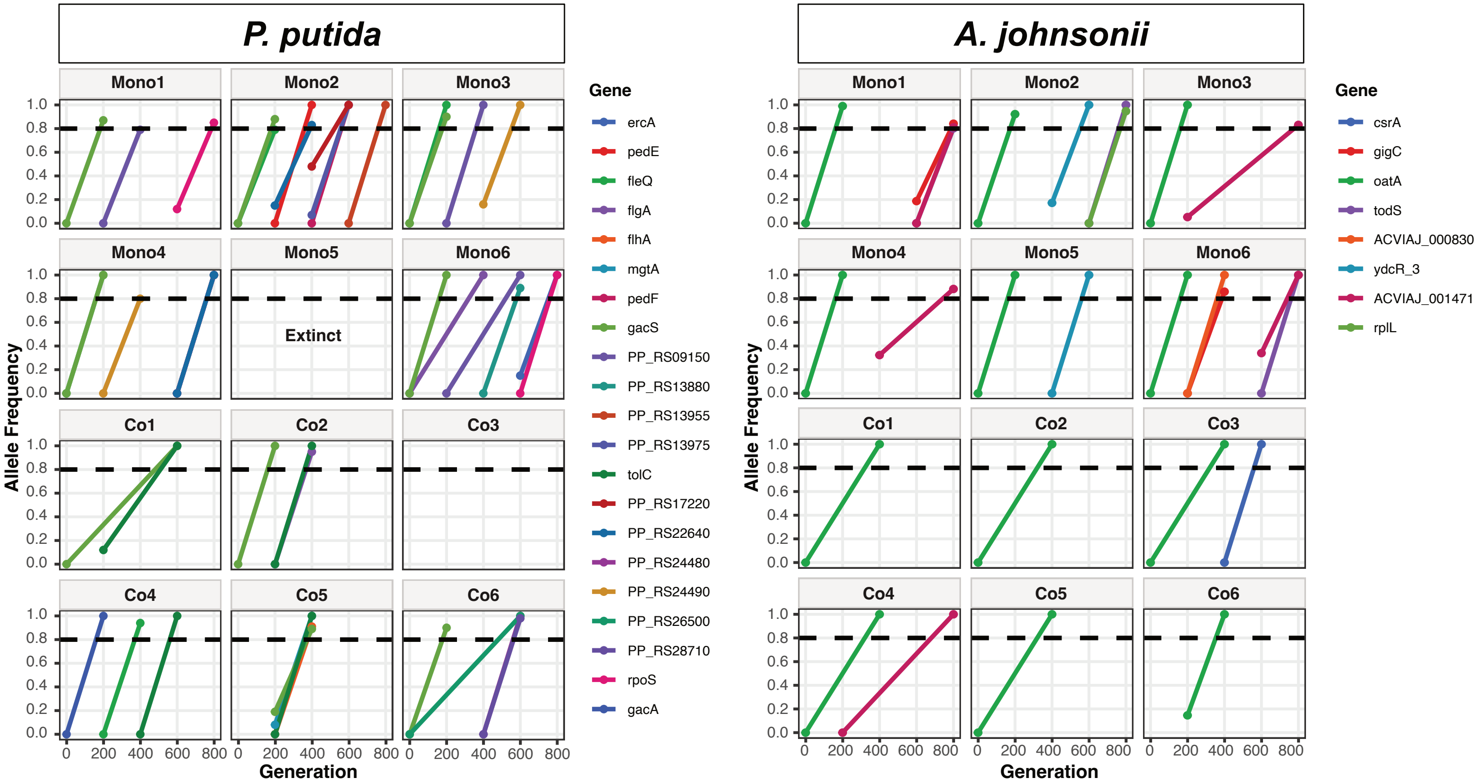
P. putida
A. johnsonii
Mono1
Mono2
Mono3
Mono4
Mono5
Mono6
Extinct
Co1
Co2
Co3
Co4
Co5
Co6
Allele Frequency
Generation
Gene
ercA
pedE
fleQ
flgA
flhA
mgtA
pedF
gacS
PP_RS09150
PP_RS13880
PP_RS13955
PP_RS13975
tolC
PP_RS17220
PP_RS22640
PP_RS24480
PP_RS24490
PP_RS26500
PP_RS28710
rpoS
gacA
csrA
gigC
oatA
todS
ACVIAJ_000830
ydcR_3
ACVIAJ_001471
rplL
